# On the Cutaneous Eruptions Induced by Various Medical Substances—Opium

**Published:** 1848-01

**Authors:** 


*On the Cutaneous Eruptions Induced by Various Medical Substances.—Opium.* The eruptions which in certain individuals follow the use of the preparations of opium are always of an exanthematous nature. In general they consist of red isolated patches not unlike those of measles. This kind of eruption is rare.

The *solaneæ.*—The eruption induced by the ingestion of the preparations of this tribe of plants are also of the order exanthemata, and are as uncommon as those which are the effect of opium. The patches are larger and irregular, resembling scarlatina.

The *oleo-resins.*—All the medical substances of this class are liable to be followed by cutaneous eruptions, but none so frequently as turpentine and copaiba. The eruption very much resembles that produced by opium and belladonna, being sometimes measly, at other times scarlatinous in its appearance. It is a rare exception to see either vesicles, pustules, or papules.

*Cod-liver Oil.*—This medicine sometimes gives rise to a form of eczema, which appears generally about the fifth day from the commencement of its use; it is however, rarely observed.

*Iodide of Potassium.*—The eruptions which follow the use of this medicine are far from uniform, sometimes being eczematous, at others, pustular, as in acne. It sometimes happens that the skin escapes the action of the medicine, and that the mucous membranes are attacked instead; in such cases as we observe coryza and conjunctivitis, which cease as soon as its use is suspended, but which will not yield to topical treatment as long as the medicine is persisted in.

The discrimination of the cutaneous affections which are induced by different medicinal substances taken internally, is of no slight practical importance; we have seen ignorance of these characters and causes give rise to unpleasant mistakes.—*From Annuaire de Therapeutique, in Provincial Journal.—Buffalo Med. Jour. and Monthly Rev.*

